# Effect of the joint fermentation of pyracantha powder and glutinous rice on the physicochemical characterization and functional evaluation of rice wine

**DOI:** 10.1002/fsn3.2560

**Published:** 2021-09-04

**Authors:** Xiaoyu Wang, Huanyi Yang, Rungang Tian, Yiwei Mo, Lijia Dong, Chi Shen, Xueyuan Han

**Affiliations:** ^1^ School of Life Science Shaoxing University Shaoxing China

**Keywords:** antioxidant, pyracantha, rice wine, volatile compounds

## Abstract

*Pyracantha fortuneana*, as a kind of wild plant resource for both medicine and food, has high nutrition and health‐care value. This study was to explore the effect of the joint fermentation of pyracantha powder and glutinous rice on the physicochemical and functional characterization of rice wine, aiming to improve the rice wine functional quality. As a result, a light dry rice wine fermented with *P. fortuneana* (PRW) was obtained using the fermentation technology of the Chinese rice wine. Although the contents of alcohol and protein in PRW were lower compared with the rice wine (RW) without adding pyracantha powder, the contents of sugar, ascorbic acid, total phenols, total flavonoids, and anthocyanins were higher in PRW. The analysis of volatile compounds by GC‐IMS showed that the contents of most aldehydes, alcohols, and esters increased in PRW. The quantification of organic acids and phenolic monomers indicated that most of the monomers determined were more abundant in PRW. Besides, the antioxidant capacity of PRW, including the scavenging rate of DPPH• and ABTS^+^•, was significantly stronger than that of RW. The bacteriostatic effect of the phenolic extracts from PRW was also observed obviously. It was expected to provide an effective way for the comprehensive utilization of *P. fortuneana* resource by producing a kind of nutritious and healthy pyracantha rice wine.

## Graphical abstract

By evaluating physicochemical properties, sensory evaluation, antioxidant, and bacteriostatic capacity and chemical compositions of pyracantha rice wine (PRW) and rice wine (RW), it was demonstrated that it was feasible to ferment glutinous rice jointly with the dry powder of pyracantha fruit to produce PRW, which gave rice wine new typicality from sense and function. Although the alcohol content of PRW was lower compared with RW, PRW was superior to RW in terms of the contents of total phenols, total flavonoids and anthocyanins, the compositions and relative amounts of volatile compounds, further the antioxidant and bacteriostatic effects.

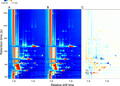

## 1 INTRODUCTION

*Pyracantha fortuneana*, belonging to the Rosaceae family, is an Asian species of the genus *Pyracantha*, and mainly distributes in China and Japan. From September to February, *P. fortuneana* sets small orange or scarlet fruits throughout the southern and northwestern in China. In the past, the fruits were ground into flour by the local people and used as food substitutes during the period of food shortage. *P. fortuneana* fruit (PFF) is rich in protein and starch, and has certain health benefits. The medicinal value of PFF was recorded by Li Shizhen in ‘‘Pen Tsao Kang Mu’’ in Ming Dynasty, which described the function of PFF in promoting metabolism and circulation, anti‐ageing, and whitening skin. PFF has been approved as a new food source by the Ministry of National Health of China. However, PFF is rarely consumed in their original state due to their less juicy, seedy, and astringent. It is a reasonable strategy to process fresh or dried PFF into high‐value products for food industries.

Studies have shown that PFF has good medicinal and edible value (Hou et al., 2003; Hou et al., 2003). PFF is a traditional Chinese medicine used to treat indigestion (Wei et al., 2017). Recent studies have shown that PFF possesses significant antioxidant activity and may promote lipoprotein metabolism in rats (Hou et al., 2002). The antioxidant, immune, and anti‐tumor effects of PFF polysaccharides have been demonstrated in mice by Yuan et al. (2010) and Yao et al. (2020). Besides, it is also used as a skin‐whitening agent in cosmetics in Japan, possibly functioning by inhibiting tyrosinase (Rong & XiaoHong, 2015).

As a kind of wild plant resource for both medicine and food, PFF is rich in nutrients, such as vitamins, amino acids, and beneficial microelements, as well as anthocyanins, polyphenols, and other flavonoids which are beneficial to the human body (Jiang et al., 2013). However, there were few reports about alcoholic beverage related to pyracantha. The present pyracantha‐related alcoholic beverage was usually made by blending fresh pyracantha juice with liqueur. And there was no report on fermented pyracantha wine. The production of fermented pyracantha wine with health‐care function would be attractive. At present, fruit wine production mostly adopts the traditional soaking or blending method. This kind of wines was not outstanding in balanced flavor. Due to the high content of organic acids and less sugar and water in PFF, the wine brewed directly from PFF juice has drawbacks, such as over acid and poor taste coordination. Therefore, the dry powder of PFF and steamed glutinous rice were fermented together using the fermentation technology of Chinese rice wine, in order to improve the quality of pyracantha wine in present study.

Shi et al. (2013) added the radix puerariae into glutinous rice to brew a new‐type nutritional rice wine with uncooked materials. Similar studies on the use of medicinal and food homologous materials in rice wine fermentation were few. In this study, the dried fruit of *P. fortuneana* was used for fermentation to produce pyracantha rice wine, which, on the one hand, promoted the comprehensive utilization of *P. fortuneana*, and on the other hand, tried to improve the functionality and quality of traditional rice wine.

## 2 MATERIALS AND METHODS

### 2.1 Preparation of *P. fortuneana*

*Pyracantha fortuneana* dried fruit was purchased from Dujiangyan county, Sichuan province of China. After removing impurities and rotten fruits, sterilization was carried out at 121℃ for 15 min in an autoclave. Then, the dried fruit was pulverized, and the powder was screened through a 60‐mesh sieve.

### 2.2 Fermentation

Pyracantha rice wine (PRW). The fermentation was carried out in a 2.0 L fermentor containing 0.6 kg steamed glutinous rice, 1.0 L distilled water, 80 g fruit powder of *P. fortuneana*, 0.08 g nisin, and 1.35 g glucoamylase (100,000 U g^−1^, Jiangsu Ruiyang Biotech co. Ltd.). Then, the activated yeast (*Saccharomyces cerevisiae*, BV818 from Angel Yeast Co., Ltd.) of 0.32 g, approximately 0.2 g/kg of the total fermentation weight, was added to the mixture. Following, the fermentor with a total weight of about 1.68 kg was kept at 24±1℃. The content of total soluble matter (°Bx) was measured using a digital refractometer every day during fermentation. When the content of total soluble matter reached stable and no bubble produced in the fermentor, it was reaching the end of fermentation. After fermentation completing on the 10th day, the wine was racked, properly clarified, and then sterilized.

Rice wine (RW). The other fermentation procedures were the same as those of the PRW, except that the PFF powder was not added.

### 2.3 Measure of physicochemical properties

By means of rapid distillation apparatus (Super DEE, GIBERTINI, Italy) and alcohol tester (AlcoMat‐2, GIBERTINI, Italy), the alcohol concentration was measured by the distillation‐alcoholmeter method. The content of total sugar was determined by employing the protocol of DNS method (Miller, 1959), and glucose solutions were prepared to make a calibration curve spectrophotometrically. According to the method of Association of Office Analytical Chemists (1996), the ascorbic acid content was measured by 2, 6‐dichlorophenol‐indophenol titration method with L‐ascorbic acid as the standard. The contents of total acid and amino acid nitrogen were measured by the method of titratable acidity according to the State Standard of the People's Republic of China GB/T 2018 (Huangjiu) and expressed in terms of tartaric acid equivalent (g/L). Using bovine serum albumin (BSA) as the standard, the total protein content was determined according to the Bradford method (Bradford, 1976).

Total anthocyanin content was determined by the extinction coefficient method (Edgar et al., 2011). Wine sample was mixed with acidified ethanol (1.5 mol/L: 95% ethanol = 15:85) and determined the absorbance value at 520 nm after keeping in the dark for 30 min. The result was calculated with cyanidin‐3‐glucoside as follows:

$$\text{Cyanidin}‐3‐\text{glucoside}\left({\text{mg L}}^{‐1}\right)=\frac{\left(A\times M_{W}\times \text{DF}\times 1000\right)}{\varepsilon }$$

Note: M_W_: molecular weight of cyanidin‐3‐glucoside (449.2).

Ɛ: molar extinction coefficient (26,900).

DF: dilution multiple of the sample.

### 2.4 Sensory evaluation

The sensory characteristics of wine samples were evaluated in terms of taste/flavor (sweet, sour, and bitter), appearance (color and turbidity), mouth‐feeling (astringency, continuation, and full body), and aroma (alcohol, fruit, and cereal) by 10 experienced panelists (five males and five females). In a sensory evaluation room of 20 ± 1℃, wine samples were evaluated in the same opaque disposable plastic cup, and each sample was repeated three times. All samples were randomly and individually presented to the panelists. Quantification of each sensory descriptor was expressed using intensity ratings ranging from 0 to 9 (0: none; 1–2: very weak; 3–4: ordinary; 5–6: moderate; 7–8: intensity; 9: high intensity) (Jung et al., 2014; Yang et al., 2017).

### 2.5 Volatile compounds determination

A FlavourSpec^®^ static headspace (SHS) gas chromatography–ion mobility spectrometry (GC‐IMS) instrument (Gesellschaft für Analytische Sensorsysteme mbH (G.A.S.), Dortmund, Germany), equipped with an MXT‐WAX (capillary column 30 m × 0.53 mm ID, 1 µm) (CS‐Chromatographie Service GmbH, Düren, Germany), a splitless injector, and an autosampler (PAL RSI, CTC Analytics AG, Zwingen, Switzerland), was employed to analyze volatile compounds.

In accordance with the method of Han, Peng, et al. (2020), the volatile compounds of wine samples were detected. Wine sample of 1 ml was placed in a 20‐mL headspace bottle and incubated at 60℃ for 10 min. The analysis parameters of GC‐IMS were as follows: total analysis time 30 min, column temperature 60℃, carrier gas N_2_, IMS temperature 45℃, injection volume 100 µl, syringe temperature 85℃. The detailed programs of ionization and drifting were according to Han, Peng, et al. (2020).

Under positive mode, IMS data were collected and evaluated using VOCal software (G.A.S.), including Dynamic PCA plugins, Gallery Plot and the Reporter. The software of GC×IMS Library Search^®^ (G.A.S.) based on NIST and IMS database was employed to identify compounds. Further, the double separation obtained in the GC column and IMS drift tube was shown in a topographic plot, where each feature was defined by retention time, drift time, and intensity value. In addition, the compound was quantified with 4‐ methyl −2‐ amyl alcohol as the internal standard.

### 2.6 Determination of phenolic monomers

Identification and quantification of phenolic monomers, including caffeic acid, chlorogenic acid, protocatechuic acid, ferulic acid, catechin, quercetin, rutin, p‐coumaric acid, gallic acid, epicatechin, and cyanin‐3‐glucoside, were implemented by HPLC analysis. First, the pH value of wine samples of 15 ml was adjusted to about 7 using 1 mol/L NaOH. Then extract with ethyl acetate for three times and mix the supernatant (this was neutral phenols). Adjust the pH of the remaining part to about 2 using 2 mol/L HCL. The following operations were the same as the above (this was acidic phenols). Neutral and acidic phenols were mixed and dried by rotary evaporation at 45℃. The residue was dissolved in 4‐ml methanol and filtered with 0.45‐µm microporous filter for HPLC analysis.

The HPLC analysis was performed on a Shimadzu LC‐20A HPLC system (Shimadzu Corporation) equipped with a Shim‐pack GIST C_18_ column (5 μm, 4.6 I.D. × 250 mm). The procedure of gradient elution was performed according to the following: solvent A, 1% (m/v) acetic acid aqueous solution; solvent B, methanol; 0–10 min, 5%–30% B, 95%–70% A; 10–25 min, 30%–50% B, 70%–50% A; 25–30 min, 50%–60% B, 50%–40% A; 30–35 min, 60%–70% B, 40%–30% A. The column temperature was 19℃, flow rate 1 ml/min, detection wavelength 280 nm, and injection volume 20 μl. Identification of compounds was achieved by comparing their retention time with standard substances. The quantification of phenolic monomers was calculated based on the standard curve of concentration and peak area of each standard substance by external standard method.

### 2.7 Measure of total flavonoids, total phenols, and antioxidant capacity

The contents of total flavonoids and total phenols in wine samples were determined based on the method of Jia et al. (1999) and Folin‐Ciocalteu (Singleton & Rossi, 1965), respectively. The content of total flavonoids was expressed as rutin equivalents (RE)/ml wine and the content of total phenols as gallic acid equivalent (GAE)/mL wine. The detailed experimental procedures referred to Han, Zhao, et al. (2020).

The scavenging capacity of DPPH (1, 1‐diphenyl‐2‐picrylhydrazyl) radical and ABTS^+^ (2, 2′‐azino‐bis (3‐ethylbenzothiazoline‐6‐sulfonic acid)) radical was determined based on the method of Brand‐Williams et al. (1995) and Re et al. (1999), respectively. The implementation details were according to Han, Zhao, et al. (2020). The radical scavenging capacity of ascorbic acid standard solution was taken as a reference. The radical scavenging rate was calculated by the following formula:

$$\text{Radical scavenging rate}\left(\%\right)=\left[1‐\frac{\left(A_{S}‐A_{0}\right)}{A_{b}}\right]\times 100$$

Note: A_S_, absorbance of the mixture of wine sample and DPPH• or ABTS^+^• solution; A_0_, absorbance of the mixture of wine sample and 95% ethanol; A_b_, absorbance of the mixture of distilled water and DPPH• or ABTS^+^• solution.

The determination of total antioxidant capacity was carried out referring to our previous paper [17]. In detail, total antioxidant capacity was measured by use of the total antioxidant capacity (T‐AOC) kit (Nanjing Jiancheng Bioengineering Institute, Nanjing, China). The measurement principle was ferric ion reducing antioxidant power (FRAP), which was that Fe^3+^ could be reduced to Fe^2+^ by antioxidants under acidic conditions, and Fe^2+^ can form a stable complex with phenanthrolines. Then, the absorbance was determined at 520 nm,

$$T‐\text{AOC}\left(U/\text{mL}\right)=\frac{OD_{M}‐OD_{C}}{0.01\times 30}\times N\times n$$

Note: OD_M_, the absorbance of the measuring tube; OD_C_, the absorbance of the control tube; N, dilution multiple of reaction system (total volume of reaction solution/sampling volume); n, dilution multiple before sample testing.

### 2.8 Determination of the bacteriostatic ability

Three foodborne pathogenic bacteria strains, including *Staphylococcus aureus* (CICC 10,001), *Escherichia coli* (CICC 10,899), and *Salmonella typhimurium* (CICC 10,420) purchased from China Center of Industrial Culture Collection (http://www.china‐cicc.org/), were used to evaluate the bacteriostatic ability of PRW. Briefly, 0.1 ml of about 10^6^ CFU/mL (OD 610 nm 0.13–0.15) bacterial cultures was evenly spread on lysogeny broth (LB) agar plate. After that, a round puncher (6 mm in diameter) was used to punch holes on the plate. The 50 µl of RW, PRW, 10% ethanol solution, and phenolic extracts from PRW were, respectively, transferred into the holes. Before transferring, all test samples were filtered sterilized by 0.22 µm microporous filter. After incubating for 24 h at 37℃, the inhibition zone formed around the hole was observed.

### 2.9 Statistic analysis

All experiments were carried out with at least three biological replicates. Data were presented as the average value ± standard deviation. The statistical significance of differences was evaluated by least‐significant difference (LSD) test on SPSS version 20.0 software. Differences were considered statistically significant at *p* < .05 and *p* < .01, and expressed with “*” or “**”, respectively.

## 3 RESULTS AND DISCUSSION

### 3.1 Basic physicochemical properties and sensory evaluation

The basic physicochemical propertied of RW (the control) and PRW were determined and are displayed in Table 1. Because of the content of total sugar <15.0 g/L, nonsugar soluble solids >5.0 g/L, alcohol >6% (v/v), total acid (tartaric acid equivalent) 1.9–5.4 g/L, amino nitrogen >0.2 g/L, both wine samples of RW and PRW were classified as light dry rice wine according to the State Standard of the People's Republic of China GB/T 2018 (Huangjiu). The levels of total sugar and nonsugar soluble solids in PRW were slightly higher than that in RW. It was considered to be due to the amount of sugar in PFF itself. In aspect of the contents of total protein, ascorbic acid, total phenols, total flavonoids, and total anthocyanins, significant differences were observed between RW and PRW. The total protein content in RW was more than twice that of PRW. However, the contents of ascorbic acid, total phenols, flavonoids, and anthocyanins of PRW were much higher than that of RW, especially the content of total flavonoids was nearly four times higher.

**TABLE 1:** Physicochemical properties of wine samples

Characteristics	RW	PRW
Total sugar (g/L)	1.86 ± 0.02	4.77 ± 0.07
Total acid (g/L)	2.10 ± 0.16	2.75 ± 0.29
Nonsugar soluble solids (%)	5.70 ± 0.01	6.43 ± 0.06
Alcohol content (%(v/v))	9.96 ± 0.62	8.48 ± 0.14
Amino nitrogen (g/L)	0.48 ± 0.01	0.46 ± 0.03
Reducing sugar (g/L)	1.75 ± 0.05	2.20 ± 0.07
Total protein (mg/L)	261.74 ± 10.61	120.10 ± 9.87
Ascorbic acid (g/L)	5.15 ± 0.04	9.86 ± 0.78
Total phenols (mg GAE/L)	195.71 ± 6.04	249.85 ± 4.71
Total flavonoids (mg RE/L)	24.21 ± 0.01	88.50 ± 8.24
Total anthocyanins (mg/L)	0.15 ± 0.09	0.71 ± 0.06

Polyphenols are important part of the composition basis of fruit health benefits (van Dorsten et al., 2010; Ohgidani et al., 2012). It was reported that phenolic extracts from different origins, which had the potential to control malolactic fermentation and potential applicability as a partial alternative to sulfur dioxide during the wine aging (García‐Ruiz et al., 2012; González‐Rompinelli et al., 2013). Anthocyanidins had the anti‐inflammatory properties and the main monomer of PFF anthocyanin was cyanin‐3‐glucoside (Ignat et al., 2011; Wei et al., 2017). Compared with RW, it is reasonable to consider PRW with higher health‐care effect, due to its significantly higher levels of total flavonoids, total phenols, and total anthocyanins content.

**FIGURE 1:**
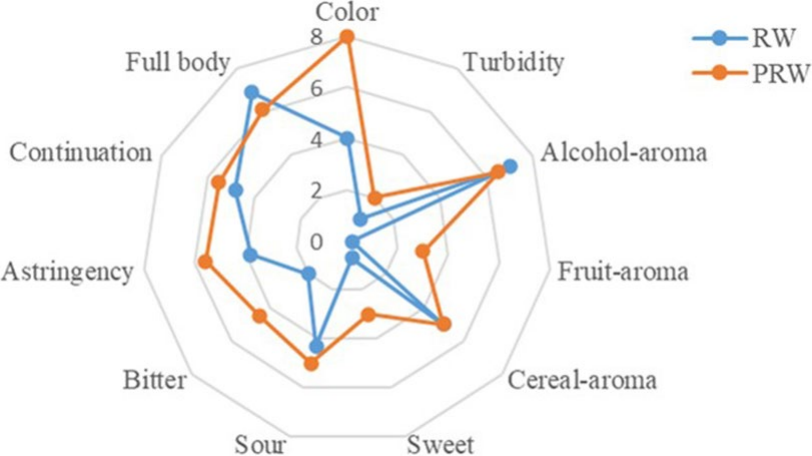
Sensory evaluation of wine samples (PRW, pyracantha rice wine; RW, rice wine)

Sensory evaluation is an important tool to evaluate product quality comprehensively and is the basis of consumer acceptability. The sensory evaluation showed that wine samples of RW were light yellow, clear and transparent, elegant, and no abnormal flavor, mellow taste, body coordination. The detailed comparison is illustrated in Figure 1. Compared with RW, PRW was light red, bright color, overall smell coordination with fruit aroma, mellow and refreshing taste, a little sweet, obvious astringency, full‐body, the aftertaste with a unique style of pyracantha fruit.

### 3.2 Volatile compounds determination

**FIGURE 2:**
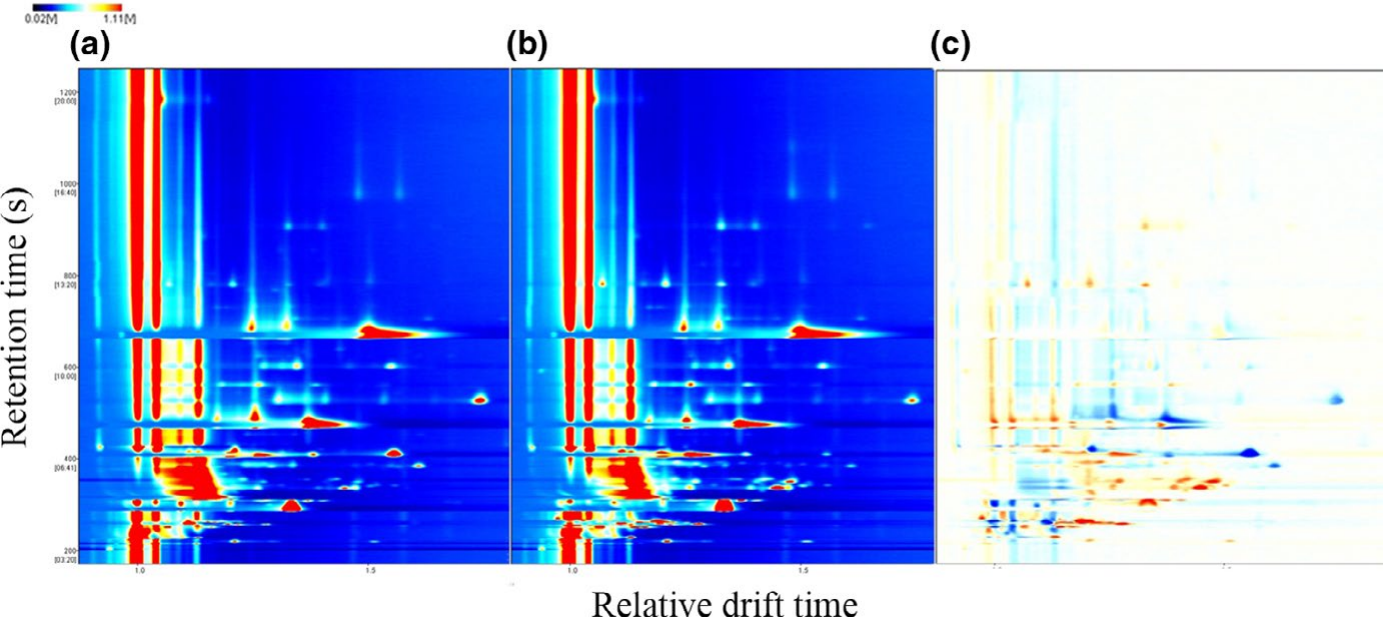
The top views of topographic plots of GC‐IMS chromatogram (A, RW; B, PRW; C, PRW deducted with RW)

The flavor is the most essential characteristic of an alcoholic beverage. The difference in volatile compounds between RW and PRW was analyzed by GC‐IMS. GC‐IMS technique is especially suitable for trace analysis of volatile organic compounds and has the advantages of accuracy, rapidity, and no sample pretreatment (nondestructive). It combines the advantages of high sensitivity and the fast response of ion mobility spectrometry with the high separation efficiency of gas chromatography. A three‐dimensional topographic plot, including retention time, migration time, and peak intensity of volatile compounds was obtained (Figure S1). The difference in volatile compounds between different groups can be seen directly from the topographic plot. However, for further observation, the top view was taken for comparison (Figure 2a, b). To compare this difference more obviously, the chromatogram of RW was selected as a reference, and the chromatogram of PRW was deducted with the reference, and the difference comparison mode was used for analysis (Figure 2c). If the compounds in the two samples were the same, the background after deduction was white, while blue indicated that the concentration of the compound was lower than the reference, and red indicated that the concentration of the compound was higher than that of the reference. As can be seen from the difference comparison graph (Figure 2c), the small molecule volatile compounds in PRW increased (chromatogram bottom), while the relatively large molecule volatile compounds decreased (chromatogram top).

**FIGURE 3:**
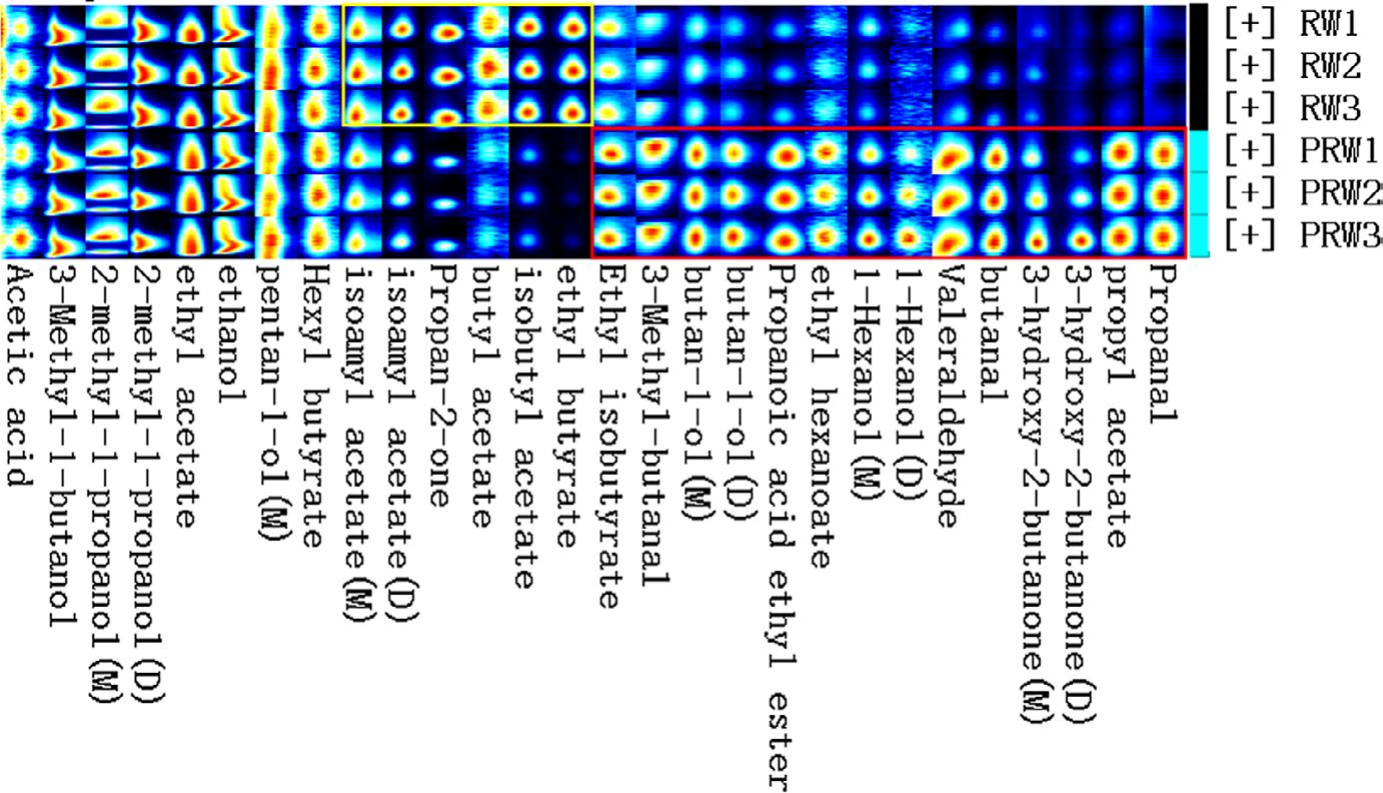
Fingerprint plot of the peaks of the identified compounds

Through the database searching, the compounds were identified (Table 2, Figure S2), and the peaks of the identified compounds were compared and analyzed by fingerprint (Figure 3) (no displaying those compounds not identified). Each compound was also quantified by the internal standard method (dimer preferred). From the result, the contents of most aldehydes, alcohols, and esters with carbon chain length less than 6 were higher in PRW, including propanal, propyl acetate, acetoin, butyraldehyde, glutaraldehyde, 1‐hexanol, ethyl caproate, ethyl propionate, 1‐butanol, 3‐methylbutyraldehyde, and ethyl isobutyrate, etc. However, the content of some esters with carbon chain length more than 6, including ethyl butyrate, isobutyl acetate, butyl acetate, and isoamyl acetate, decreased in PRW. The integral parameters of each identified volatile compound are detailed in Table S1.

**TABLE 2:** Qualitative and quantitative determination of volatile compounds by GC‐IMS

	Compound	CAS#	Formula	Comment	Content (µg/L) in RW	Content (µg/L) in PRW
1	Acetic acid	C64197	C2H4O2		81.65	92.02
2	1‐Hexanol	C111273	C6H14O	Monomer		
3	1‐Hexanol	C111273	C6H14O	Dimer	23.85	41.74
4	3‐Methyl−1‐butanol	C123513	C5H12O		6,023.05	6,702.27
5	2‐methyl−1‐propanol	C78831	C4H10O	Monomer		
6	2‐methyl−1‐propanol	C78831	C4H10O	Dimer	4,520.05	3,823.95
7	ethyl acetate	C141786	C4H8O2		4,311.51	6,137.28
8	ethanol	C64175	C2H6O		4,823.69	5,233.74
9	pentan−1‐ol	C71410	C5H12O			
10	ethyl hexanoate	C123660	C8H16O2		14.42	23.75
11	4‐methyl−2‐pentanol	C108112	C6H14O	Monomer		92.02
12	4‐methyl−2‐pentanol	C108112	C6H14O	Dimer	198.00	198.00
13	butan−1‐ol	C71363	C4H10O	Monomer		
14	butan−1‐ol	C71363	C4H10O	Dimer	61.60	120.21
15	isoamyl acetate	C123922	C7H14O2	Monomer		
16	isoamyl acetate	C123922	C7H14O2	Dimer	705.54	496.64
17	isobutyl acetate	C110190	C6H12O2		208.28	87.35
18	ethyl butyrate	C105544	C6H12O2		1,185.25	129.30
19	Propanoic acid ethyl ester	C105373	C5H10O2		198.19	648.62
20	propyl acetate	C109604	C5H10O2		52.40	265.18
21	Propan−2‐one	C67641	C3H6O		808.02	408.45
22	Hexyl butyrate	C2639636	C10H20O2		139.42	169.29
23	3‐hydroxy−2‐butanone	C513860	C4H8O2	Monomer		
24	3‐hydroxy−2‐butanone	C513860	C4H8O2	Dimer	73.05	190.70
25	butyl acetate	C123864	C6H12O2		22.73	15.58
26	Valeraldehyde	C110623	C5H10O		48.56	146.04
27	Ethyl isobutyrate	C97621	C6H12O2		20.19	34.80
28	3‐Methyl‐butanal	C590863	C5H10O		37.11	67.57
29	Propanal	C123386	C3H6O		70.11	216.71
30	butanal	C123728	C4H8O		57.97	289.08

**FIGURE 4:**
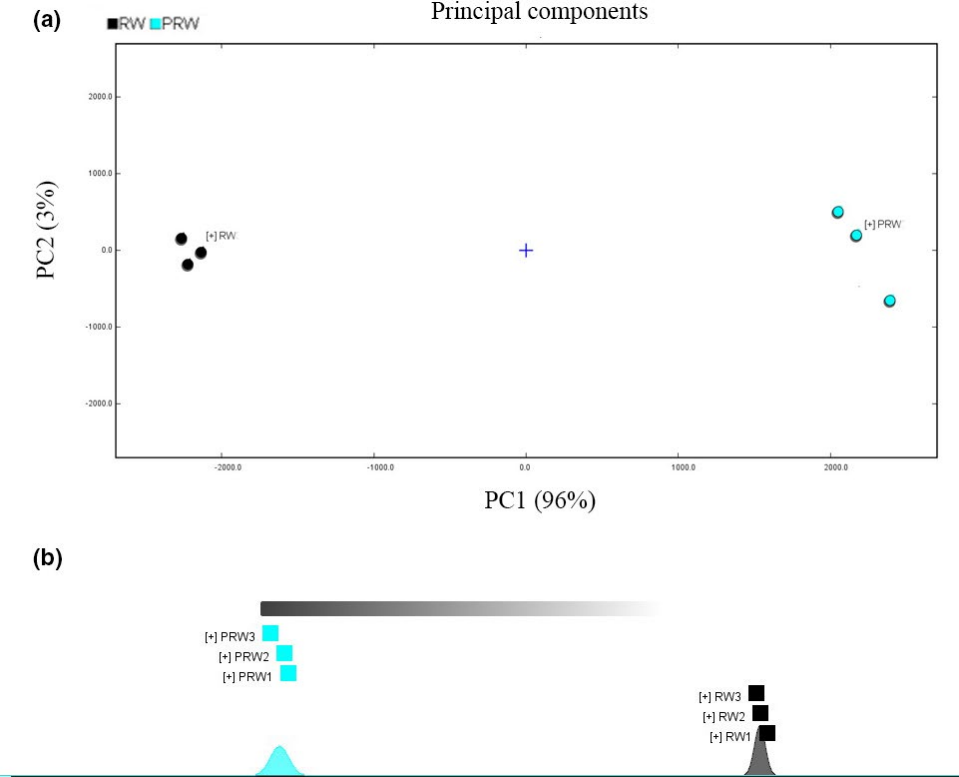
Clustergram of principal component analysis (PCA) and fingerprint similarity analysis based on the peak volume of each compound

Furthermore, the principal component analysis (PCA) and fingerprint similarity analysis based on the peak volume of each compound showed that the repeatability of the experiment was well, and the composition and content of volatile compounds were significantly different between RW and PRW (Figure 4).

A wide range of volatile compounds, including esters, aldehydes, alcohols, terpenes, furans, and benzene derivatives, were accumulated during fermentation and microbial metabolism, which gave the wine a unique style and character (Franitza et al., 2016; Hoang et al., 2016). Volatile compounds of rice wine were influenced by various factors, such as raw material, yeast starters, and fermentation conditions. Microbial metabolism and community during fermentation were closely related to and contributed to the production of volatile compounds in wine making (Medina et al., 2013; Moreira et al., 2011). Many researches have focused on yeast development that produces a lot of flavor compounds (Gobbi et al., 2013; Maturano et al., 2012). It was reported that the effect of different mold starter on the aroma of rice wine was mainly reflected in the content of alcohols and esters (S. Liu et al., 2019). Besides, the addition of plant polyphenolic extracts affected the volatile composition of the wine, notably resulting in a higher content of esters and a lower content of furan compounds (González‐Rompinelli et al., 2013). The terpenoids, alkanes, alcohols, and aldehydes, most of which were precursors of aroma compounds, were determined to be dominant components in the essential oil of PFF (Wang et al., 2013). In this experiment, the volatile compounds of PRW were derived from both PFF itself and yeast metabolism that may be affected by PFF.

### 3.3 Quantification of phenolic monomers

**FIGURE 5:**
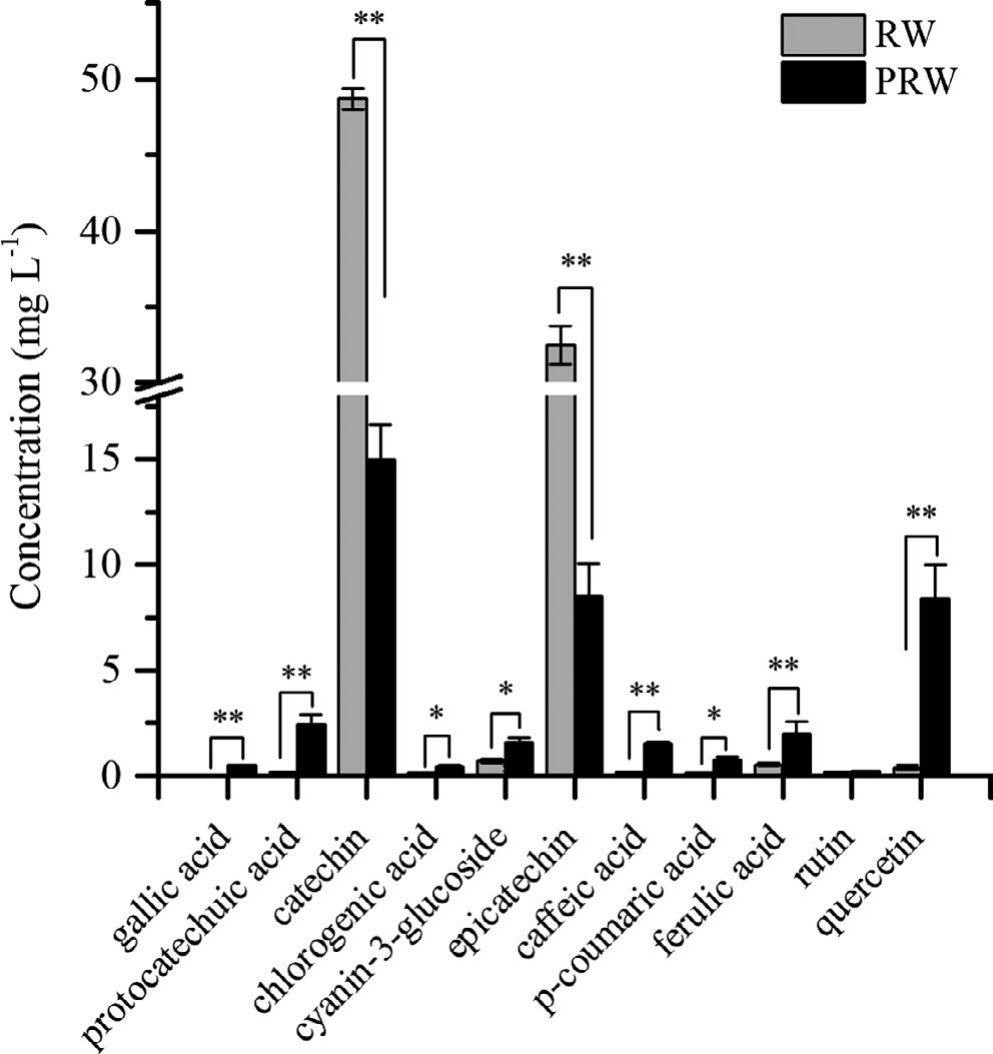
The content of phenolic monomers in RW and PRW (*p* ≤ .05, “*”; *p* ≤ .01 “**”)

To further compare the qualitative and quantitative differences in phenolic monomers, the contents of gallic acid, protocatechuic acid, catechin, chlorogenic acid, cyanin‐3‐glucoside, epicatechin, caffeic acid, p‐coumaric acid, ferulic acid, rutin, and quercetin in wine samples were determined by HPLC analysis, and the typical chromatograms are displayed in Figure S3. The standard curves of concentration and peak area of standard substances are shown in Table S2. Catechin, epicatechin, and quercetin were the main phenols, and their contents were significantly higher than that of other phenolic monomers in both wine samples (Figure 5). And the levels of catechin and epicatechin in RW, respectively, 48.69 ± 0.71 mg/L and 32.49 ± 1.27 mg/L, were significantly higher than that in PRW. Gallic acid was detected only in PRW. Although the contents of other phenolic monomers were less than catechin and epicatechin, their contents were significantly increased in PRW, except no significant difference in rutin content between RW and PRW.

In a previous study, a chemical antioxidant activity‐guided extraction was designed to obtain the optimal PFF extract, which indicated that PFF was rich in polyphenols, such as rutin, polymeric (epi)‐catechin (proanthocyanidins), and quercetin etc. (Zhao et al., 2013). The presence of polyphenolic proanthocyanidins from PFF increased the effect of quercetin on cell antioxidant activities, which suggested that the antioxidant benefits of small molecular polyphenols in vivo may be related to the additive effects of proanthocyanidins (Zhao et al., 2013). The related studies on *Pyracantha* species mainly focused on the identification or analysis of naturally active compounds, including flavonoids (Fico et al., 2000), anthocyanins (Ma et al., 2006), and other polyphenols in PFF (Dai, He, et al., 2008; Dai et al., 2008). However, according to the published data, the recovery rate of these active polyphenols was less than 0.09%, which was only a small fraction of the active chemicals in PFF. But few studies have focused on the processing and addressed the polyphenolic profiling of PFF. Through fermentation with PFF, the compositions and levels of phenolic monomers were abundant in wine samples.

### 3.4 Antioxidant capacity

**FIGURE 6:**
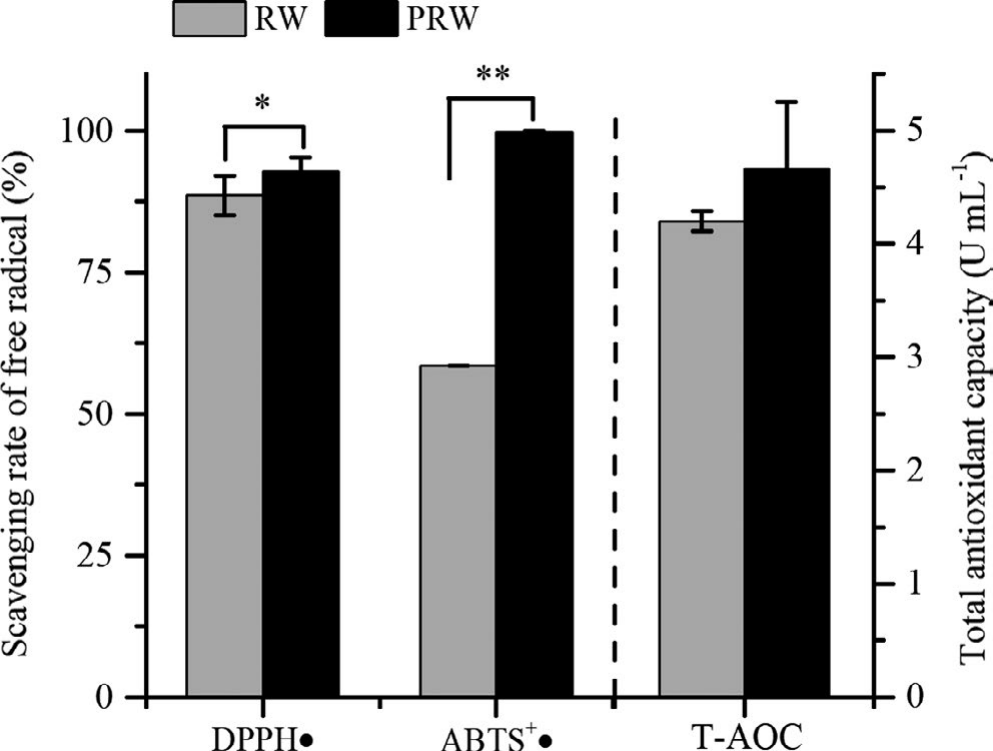
The scavenging rate of DPPH and ABTS^+^ radicals and the total antioxidant capacity (T‐AOC) (*p* ≤ .05, “*”; *p* ≤ .01 “**”)

The antioxidant capacity of wine samples was evaluated by determining the scavenging ability of DPPH and ABTS^+^ radicals and the T‐AOC based on FRAP (Figure 6). PRW presented significantly stronger scavenging rate of DPPH and ABTS^+^ radicals (more than 90%, nearly ascorbic acid equivalent 8 µg/ml) than RW. But there was no significant difference in total antioxidant capacity.

Several fruits, such as pineapple and kiwifruit, honey, and rice are used to make wine, and various kinds of herbs and spices play important roles in the production of alcoholic beverages, like enhancers, preservatives, antioxidants, and sources of antimicrobial activity (Yuwa‐Amornpitak et al., 2012). PFF is a potential preparation of therapeutic and nutritional agent (Xu et al., 2016). It was reported that PFF extract significantly decreased the levels of low‐density lipoprotein cholesterol, total cholesterol, and triacylglycerol (Xu et al., 2016). Furthermore, the amelioration of PFF extract on obesity and hyperlipidemia was closely related to the improvement of endogenous antioxidant activity (Xu et al., 2016).

Although the relationship was not entirely linear, the antioxidant capacity was directly or indirectly related to the total amount of natural polyphenols from yerba mate in vitro (Valerga et al., 2012). Besides, the complex interaction between the components of the mixture could lead to synergy, antagonism, and even the enhancement of antioxidant effect (Ray et al., 2006). For example, the synergistic antioxidant effects between caffeic acid and carnosic acid were presented, as well as quercetin and rutin (Capitani et al., 2009), further indicating that the interaction between natural phenolic compounds strongly depended on the compound proportion in the mixture and also influenced bioactivities of mixture products. The interaction between catechin and quercetin in the simulated matrix had a positive effect on the anti‐radical activity and concentration of both compounds in vitro (Turan et al., 2007). Therefore, it is difficult to determine how an individual component plays an antioxidant role.

### 3.5 Bacteriostatic effect

The bacteriostatic tests of RW and PRW were carried out in vitro by evaluating the inhibition of *Escherichia coli*, *Staphylococcus aureus*, and *Salmonella typhimurium*. The results showed that compared with RW, PRW and 10% ethanol solution, the phenolic extracts from PRW had the visible bacteriostatic effect (marked with dotted lines in Figure S4). Theoretically, the bacteriostatic test was a more biologically representative method to determine the potential biological activities of the sample than the chemistry activity assay. Although PRW presented the strong antioxidant activity, the bacteriostatic effect was not observed. Considering the visible bacteriostatic effect of the phenolic extracts from PRW, it was speculated that the lack of bacteriostatic effect might be due to the insufficient content of active ingredients in wine samples.

## 4 CONCLUSIONS

Differences in physicochemical properties, sensory evaluation, chemical compositions, antioxidant, and bacteriostatic capacity between PRW and RW were determined and analyzed. The results demonstrated that it was feasible to ferment glutinous rice jointly with the dry powder of pyracantha fruit to produce pyracantha rice wine, which gave rice wine new typicality from sense and function. Through fermenting with *P. fortuneana*, the compositions and relative amounts of volatile compounds definitely increased. The contents of phenolic monomers also presented significant differences. Furthermore, the free radical scavenging and antioxidant ability of wine samples were significantly enhanced by PFF fermentation. It was suggested that it had multiple values to develop PRW by fermentation when considering resource utilization and health‐care effect.

## CONFLICT OF INTEREST

The authors declare no competing financial interest.

## AUTHOR CONTRIBUTIONS

**Xiaoyu Wang:** Data curation (equal); Methodology (equal); Writing‐original draft (equal). **Huanyi Yang:** Formal analysis (equal); Investigation (equal); Software (equal). **Rungang Tian:** Conceptualization (equal); Resources (equal); Supervision (equal). **Yiwei Mo:** Funding acquisition (equal); Project administration (equal); Supervision (equal). **Li‐jia Dong:** Funding acquisition (equal); Project administration (equal); Supervision (equal). **Chi Shen:** Supervision (equal); Writing‐review & editing (equal). **Xueyuan Han:** Conceptualization (equal); Funding acquisition (equal); Writing‐review & editing (equal).

## Supporting information

Figure S1

Figure S2

Figure S3

Figure S4

Table S1

Table S2

## Data Availability

The data that support the findings of this study are available from the corresponding author upon reasonable request.
