# Supplementary figures and images for: Effect of the joint fermentation of pyracantha powder and glutinous rice on the physicochemical characterization and functional evaluation of rice wine
Source: Food Sci Nutr. 2021 Sep 4;9(11):6099–108. doi: 10.1002/fsn3.2560 (PMC8565233; doi:10.1002/fsn3.2560)

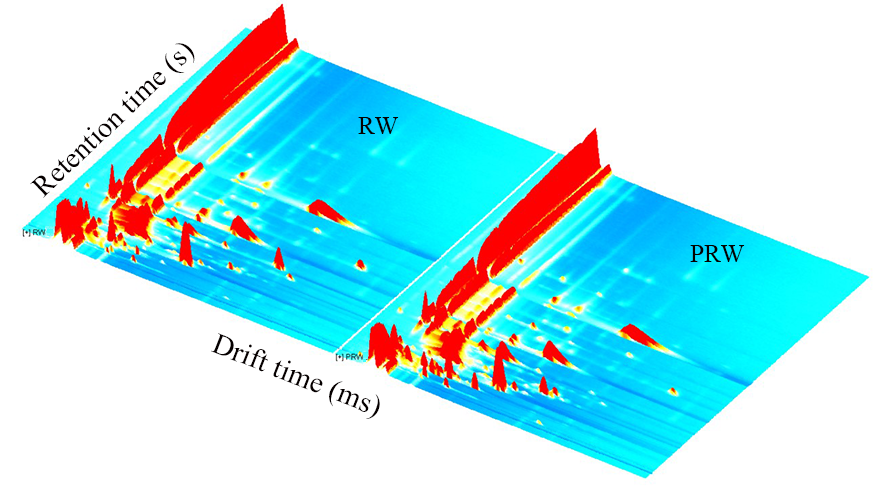

Supplement: Supplementary file 1 — Figure S1 [file FSN3-9-6099-s005.tif]

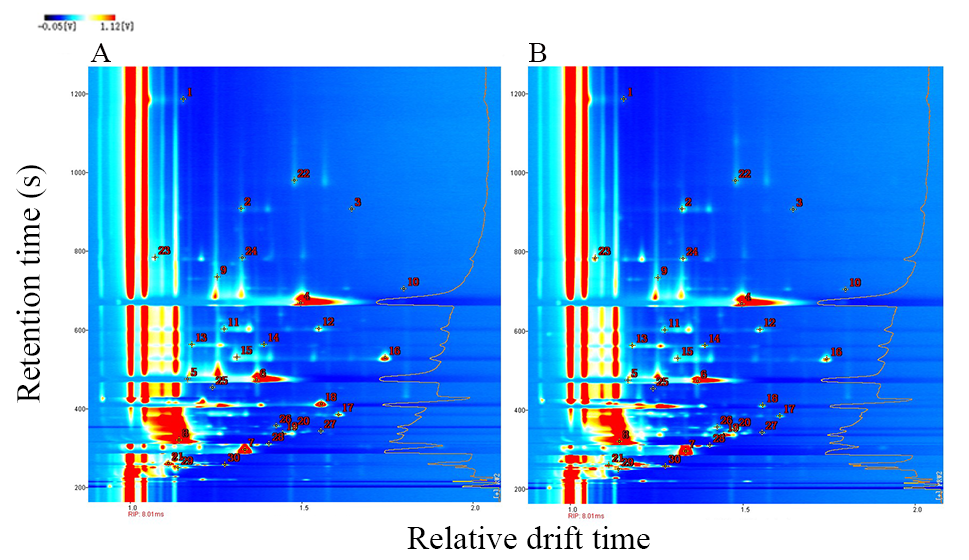

Supplement: Supplementary file 2 — Figure S2 [file FSN3-9-6099-s006.tif]

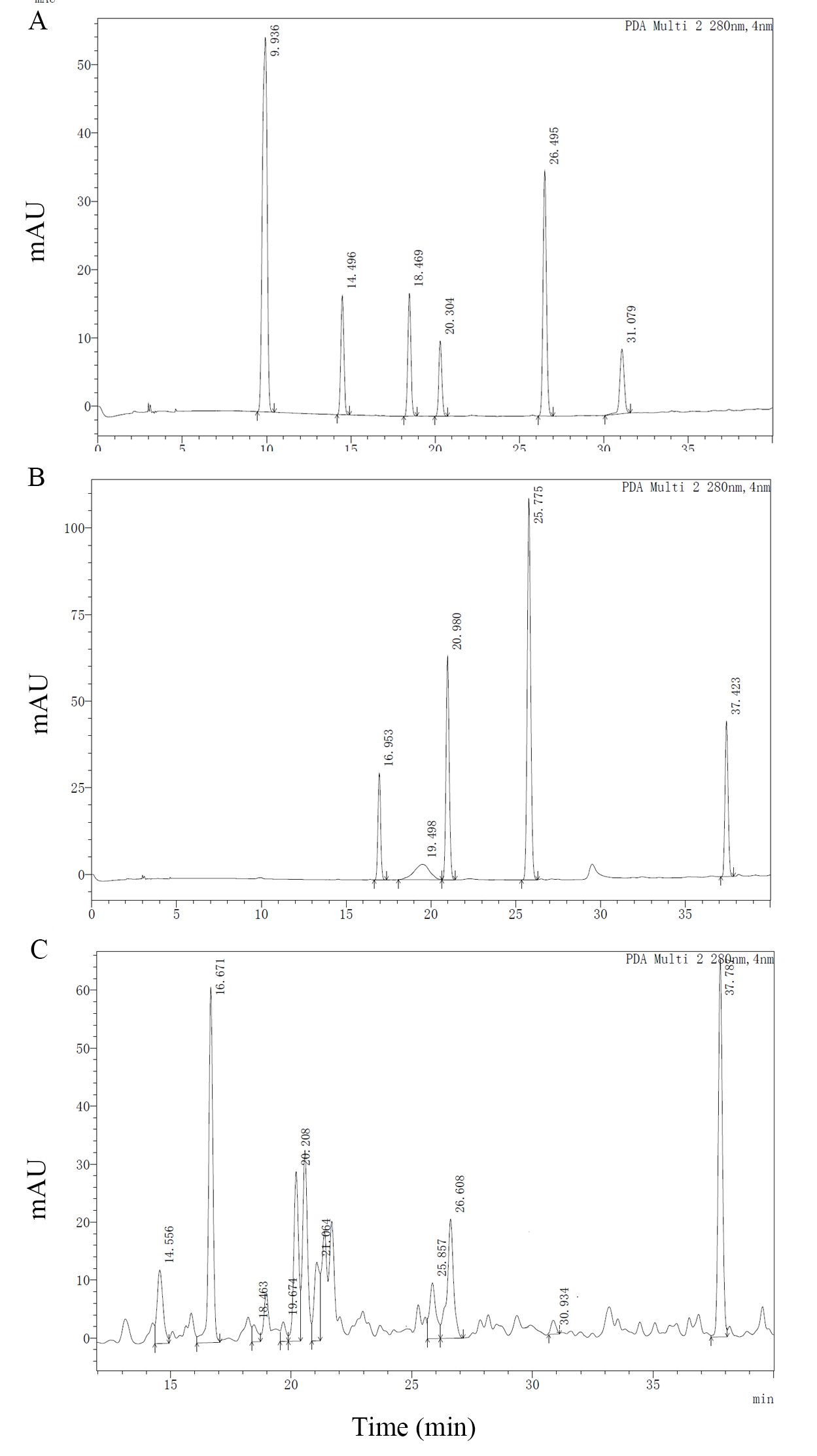

Supplement: Supplementary file 3 — Figure S3 [file FSN3-9-6099-s003.tif]

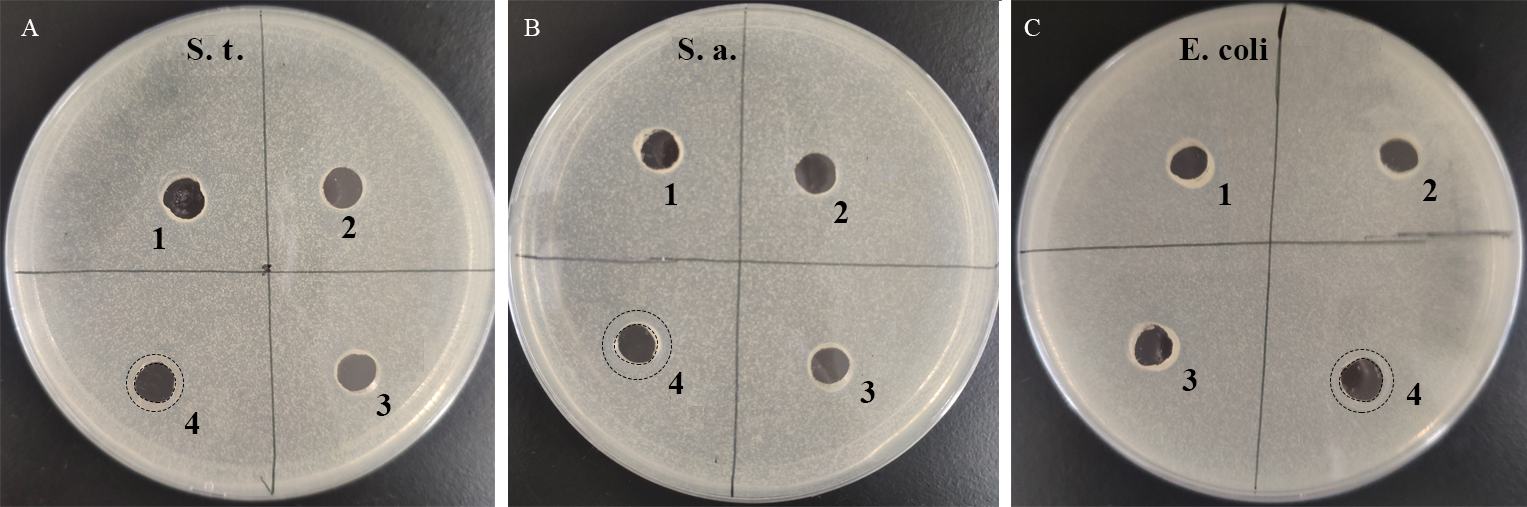

Supplement: Supplementary file 4 — Figure S4 [file FSN3-9-6099-s001.tif]
