# Supplementary material for: Effect of the joint fermentation of pyracantha powder and glutinous rice on the physicochemical characterization and functional evaluation of rice wine
Source: Food Sci Nutr. 2021 Sep 4;9(11):6099–108. doi: 10.1002/fsn3.2560 (PMC8565233; doi:10.1002/fsn3.2560)
Supplement: Supplementary file 5 — Table S1 [file FSN3-9-6099-s004.docx]

| Count | Compound | CAS# | Formula | MW | RI | Rt [sec] | Dt [a.u.] | Comment |
| --- | --- | --- | --- | --- | --- | --- | --- | --- |
| 1 | Acetic acid | C64197 | C2H4O2 | 60.1 | 1497.3 | 1186.54 | 1.1565 |  |
| 2 | 1-Hexanol | C111273 | C6H14O | 102.2 | 1367.9 | 907.966 | 1.32548 | Monomer |
| 3 | 1-Hexanol | C111273 | C6H14O | 102.2 | 1367.5 | 907.192 | 1.64857 | Dimer |
| 4 | 3-Methyl-1-butanol | C123513 | C5H12O | 88.1 | 1219.7 | 674.003 | 1.49954 |  |
| 5 | 2-methyl-1-propanol | C78831 | C4H10O | 74.1 | 1101.9 | 478.29 | 1.1686 | Monomer |
| 6 | 2-methyl-1-propanol | C78831 | C4H10O | 74.1 | 1100.1 | 475.493 | 1.37024 | Dimer |
| 7 | ethyl acetate | C141786 | C4H8O2 | 88.1 | 904.5 | 292.44 | 1.33645 |  |
| 8 | ethanol | C64175 | C2H6O | 46.1 | 946.2 | 319.146 | 1.14376 |  |
| 9 | pentan-1-ol | C71410 | C5H12O | 88.1 | 1265.5 | 734.847 | 1.25584 |  |
| 10 | ethyl hexanoate | C123660 | C8H16O2 | 144.2 | 1245.4 | 705.179 | 1.80066 |  |
| 11 | 4-methyl-2-pentanol | C108112 | C6H14O | 102.2 | 1177.2 | 602.793 | 1.27512 | Monomer |
| 12 | 4-methyl-2-pentanol | C108112 | C6H14O | 102.2 | 1177.4 | 603.191 | 1.55157 | Dimer |
| 13 | butan-1-ol | C71363 | C4H10O | 74.1 | 1156.1 | 563.734 | 1.18078 | Monomer |
| 14 | butan-1-ol | C71363 | C4H10O | 74.1 | 1155.4 | 562.538 | 1.39172 | Dimer |
| 15 | isoamyl acetate | C123922 | C7H14O2 | 130.2 | 1137.2 | 531.084 | 1.31229 | Monomer |
| 16 | isoamyl acetate | C123922 | C7H14O2 | 130.2 | 1136.0 | 529.092 | 1.74611 | Dimer |
| 17 | isobutyl acetate | C110190 | C6H12O2 | 116.2 | 1027.9 | 385.763 | 1.60996 |  |
| 18 | ethyl butyrate | C105544 | C6H12O2 | 116.2 | 1051.4 | 411.799 | 1.56014 |  |
| 19 | Propanoic acid ethyl ester | C105373 | C5H10O2 | 102.1 | 973.5 | 338.021 | 1.44616 |  |
| 20 | propyl acetate | C109604 | C5H10O2 | 102.1 | 993.8 | 351.498 | 1.48011 |  |
| 21 | Propan-2-one | C67641 | C3H6O | 58.1 | 838.4 | 260.555 | 1.11324 |  |
| 22 | Hexyl butyrate | C2639636 | C10H20O2 | 172.3 | 1405.0 | 980.25 | 1.48059 |  |
| 23 | 3-hydroxy-2-butanone | C513860 | C4H8O2 | 88.1 | 1296.8 | 783.78 | 1.07349 | Monomer |
| 24 | 3-hydroxy-2-butanone | C513860 | C4H8O2 | 88.1 | 1296.2 | 782.864 | 1.32827 | Dimer |
| 25 | butyl acetate | C123864 | C6H12O2 | 116.2 | 1086.6 | 454.141 | 1.24087 |  |
| 26 | Valeraldehyde | C110623 | C5H10O | 86.1 | 999.3 | 356.31 | 1.42737 |  |
| 27 | Ethyl isobutyrate | C97621 | C6H12O2 | 116.2 | 980.7 | 342.742 | 1.55861 |  |
| 28 | 3-Methyl-butanal | C590863 | C5H10O | 86.1 | 929.8 | 310.727 | 1.40525 |  |
| 29 | Propanal | C123386 | C3H6O | 58.1 | 818.7 | 250.887 | 1.14139 |  |
| 30 | butanal | C123728 | C4H8O | 72.1 | 834.9 | 258.836 | 1.27763 |  |

**Supplementary Table 1** GC-IMS integration parameters of volatile compounds identified in wine samples

Note: MW, molecular weight; RI, retention index; Rt, retention time; Dt: relative drift time
