# Supplementary material for: Effect of the joint fermentation of pyracantha powder and glutinous rice on the physicochemical characterization and functional evaluation of rice wine
Source: Food Sci Nutr. 2021 Sep 4;9(11):6099–108. doi: 10.1002/fsn3.2560 (PMC8565233; doi:10.1002/fsn3.2560)
Supplement: Supplementary file 6 — Table S2 [file FSN3-9-6099-s002.docx]

| Phenolic monomers | standard curve | R^2^ |
| --- | --- | --- |
| gallic acid | y = 5E+07x - 91670 | 0.9935 |
| protocatechuic acid | y = 2E+07x - 5823.2 | 0.9953 |
| catechin | y = 1E+07x - 9553.3 | 0.9938 |
| chlorogenic acid | y = 3E+07x - 10439 | 0.9964 |
| cyanin-3-glucoside | y = 1E+07x - 21680 | 0.9945 |
| epicatechin | y = 1E+07x - 7941.7 | 0.9942 |
| caffeic acid | y = 4E+07x - 20443 | 0.997 |
| p-coumaric acid | y = 6E+07x - 32752 | 0.9952 |
| ferulic acid | y = 4E+07x - 12149 | 0.9971 |
| rutin | y = 1E+07x + 6387.7 | 0.9972 |
| quercetin | y = 2E+07x - 13137 | 0.9972 |

**Supplementary Table 2** Standard curves of concentration and peak area of phenolic monomers
